# Brain-Derived Neurotrophic Factor Precursor in the Hippocampus Regulates Both Depressive and Anxiety-Like Behaviors in Rats

**DOI:** 10.3389/fpsyt.2018.00776

**Published:** 2019-01-25

**Authors:** Feng Zhong, Lei Liu, Jia-Li Wei, Zhao-Lan Hu, Li Li, Shuang Wang, Jun-Mei Xu, Xin-Fu Zhou, Chang-Qi Li, Zhao-Yun Yang, Ru-Ping Dai

**Affiliations:** ^1^Department of Anesthesiology, The Second Xiangya Hospital, Central South University, Changsha, China; ^2^Anesthesia Medical Research Center of Central South University, Changsha, China; ^3^Medical Research Center and Clinical Laboratory, Xiangya Hospital of Central South University, Changsha, China; ^4^Division of Health Sciences, School of Pharmacy and Medical Science and Sansom Institute, University of South Australia, Adelaide, SA, Australia; ^5^Department of Anatomy and Neurobiology, School of Basic Medical Science, Central South University, Changsha, China

**Keywords:** proBDNF, depression, anxiety, hippocampus, stress

## Abstract

Depression and anxiety are two affective disorders that greatly threaten the mental health of a large population worldwide. Previous studies have shown that brain-derived neurotrophic factor precursor (proBDNF) is involved in the development of depression. However, it is still elusive whether proBDNF is involved in anxiety, and if so, which brain regions of proBDNF regulate these two affective disorders. The present study aims to investigate the role of proBDNF in the hippocampus in the development of depression and anxiety. Rat models of an anxiety-like phenotype and depression-like phenotype were established by complete Freund's adjuvant intra-plantar injection and chronic restraint stress, respectively. Both rat models developed anxiety-like behaviors as determined by the open field test and elevated plus maze test. However, only rats with depression-like phenotype displayed the lower sucrose consumption in the sucrose preference test and a longer immobility time in the forced swimming test. Sholl analysis showed that the dendritic arborization of granule cells in the hippocampus was decreased in rats with depression-like phenotype but was not changed in rats with anxiety-like phenotype. In addition, synaptophysin was downregulated in the rats with depression-like phenotype but upregulated in the rats with anxiety-like phenotype. In both models, proBDNF was greatly increased in the hippocampus. Intra-hippocampal injection anti-proBDNF antibody greatly ameliorated the anxiety-like and depressive behaviors in the rats. These findings suggest that despite some behavioral and morphological differences between depression and anxiety, hippocampal proBDNF is a common mediator to regulate these two mental disorders.

## Introduction

Depression and anxiety are highly debilitating mental disorders that severely affect patients' quality of life and put a burden on families and society. Globally, depression ranks as the largest contributor to global disability and nearly 300 million people suffer from anxiety. Around half of them have comorbidity of depression and anxiety (1, 2). However, studies on depression and anxiety mechanisms and the invention of therapeutic drugs develop slowly. Clinical drugs take weeks to months to have therapeutic effects, while more than one-third of patients are still resistant to the treatment (3). Therefore, it is urgent to explore the underlying mechanism of depression and anxiety.

Brain-derived neurotropic factor (BDNF) is a member of the neurotrophin family of growth factors. It is widely expressed in different brain regions including the amygdala, hippocampus and neocortex (4). It is well known that BDNF exerts antidepressant effects in various experimental models (5–8). Notably, BDNF is first synthesized as the BDNF precursor (proBDNF), which is then intracellularly cleaved by proconvertases/furin or extracellularly processed by matrix metalloproteinases /plasmin to generate mature BDNF (9, 10). Besides being an intermediate during the synthesis of mature BDNF, proBDNF can act on its receptors and have opposite functions to mBDNF in regulating neuronal activity (11). In this regard, BDNF binds to its receptor TrkB to promote neuronal survival, differentiation and synaptic plasticity. In contrast, proBDNF induces neuronal apoptosis via activation of a receptor complex of p75 neurotrophin receptor (p75^NTR^) and sortilin (12, 13). Furthermore, proBDNF negatively regulates dendritic complexity and depresses the synaptic transmission in the hippocampus (14). Thus, proBDNF in the hippocampus may have different biological functions in anxiety/depression than mature BDNF.

Accumulating evidence has shown that proBDNF signaling is involved in the disease progress of depression. For example, clinical studies have shown that proBDNF was decreased in the postmortem cerebellum and spleen of depressed patients (15). proBDNF and its receptors p75^NTR^ and sortilin were upregulated in the serum of female depressed patients and positively correlated with depression scores (16, 17). Furthermore, the increase in proBDNF in the serum of depressed patients was reversed by long-term antidepressant treatment (16). Experimental studies have also shown that proBDNF and its receptors were increased with spine loss in the hippocampus of rats with depression, that has been induced by unpredictable, chronic, mild stress (18). Anti-proBDNF antibody (Ab-proBDNF) injection via intra-cerebroventricular and intraperitoneal approaches reversed the stress-induced depressive behavior (18). Moreover, proBDNF was upregulated in the medial prefrontal cortex while downregulated in the nucleus accumbens of learned helplessness rats (19, 20). The role of hippocampal proBDNF on regulating depressive behavior and the exact neuronal target for depression treatment are not yet clear. Moreover, the role of hippocampal proBDNF signaling in anxiety disorder is still unknown. Anxiety disorder, although sharing some symptoms with depression, is a different mental illness (21–23). In particular, anxiety usually precedes depression and eventually develops into depression (24). It was shown that hippocampal proBDNF was increased in carioca high-conditioned freezing rats, an anxiety disorder model (25). However, whether the increased hippocampal proBDNF is involved in anxiety disorders is still unknown.

This study aims to explore the possible role of hippocampal proBDNF in depression and anxiety. The rat model of anxiety was induced by complete Freund's adjuvant (CFA) intra-plantar injection and the depressed rat model was established by chronic restraint stress (CRS). The present study showed that proBDNF was upregulated in the hippocampus in these two animal models. Neutralizing the increased endogenous hippocampal proBDNF by the monoclonal Ab-proBDNF ameliorated the anxiety-like and depressive behaviors. Thus, the present study suggests that the hippocampal proBDNF is a common mediator that regulates depression and anxiety.

## Materials and Methods

Male Sprague Dawley rats (eight weeks old; weight, 250 ± 20 g) were purchased from Hunan SJA Laboratory Animal Co., Ltd. (Changsha, China). During the experiment, animals were kept in a room with a 12-h light/dark cycle and environmental temperature of 25°C with 50–60% humidity in the experimental animal center of The Second Xiangya Hospital. Rats were housed in standard cages with *ad libitum* access to food and water. All of the animals were habituated for at least 1 week before any manipulation. All of the procedures were approved by the Institution of Animal Care and Use Committee of The Second Xiangya Hospital and Use Committee, and conformed to the Guide for the Care and Use of Laboratory Animals.

### Animal Models

Depression-like behavior was induced by CRS in our previous study (26). Rats were restricted in a transparent cone made of polyvinyl chloride films. The restraint was performed 4 h per day for 3 weeks, and animals could not move except for breathing. The time of the restraint was random from 10:00 to 14:00. Then, rats were released from the cone back to the home cage after the restraint. The control rats were handled by the same experimenter without any restraints. Another group of rats was used for CFA (Sigma-Aldrich, St. Louis, MO, United States) injection. First, 100 μl CFA was injected into the left footpad to induce anxiety-like behavior as described previously (27). Three weeks later, rats were subjected to a battery of behavioral tests for the assessment of nociceptive responses, anxiety-like behaviors and depression-like behaviors.

### Nociceptive Behavioral Test

The mechanical allodynia induced by CFA intra-plantar injection was accessed by measuring the 50% paw withdrawal threshold (PWT), as described previously (28, 29). The 50% PWT in response to a series of von Frey filaments (Ugo basile, Gemonio, Italy) was determined by the up and down method. Briefly, rats were placed separately in plexiglass chambers with mesh floors for 30 min to habituate before the test. Eight von Frey filaments with bending forces ranging from 0.6 to 15 g were chosen. Firstly, the 2.0 g filament was applied perpendicular to the plantar surface of the paw for every trial. If a positive response (apparent withdrawal, licking, jumping) occurred, an “X” was recorded. Then, a weaker filament was applied. If no positive response occurred, we recorded an “O,” and a stronger filament was applied. Each trial ended when a six-number sequence of Os and Xs was obtained. The maximum and minimum limitation for filaments forces was to 0.6 and 15 g. Finally, the 50% PWT was achieved by an adjusted version of the formula presented by Chaplan (28).

### Open Field Test

Animals were placed in the center area of an open arena (120 cm long^*^120 cm wide^*^40 cm high) and were free to explore the field for 5 min. All of the movements were tracked by the overhead camera. The total travel distance, the time and travel distance in the central square (80 cm wide^*^80 cm long) were analyzed by ViewPoint Video Tracking Software (ViewPiont Behavior Technology, Lyon, France).

### Elevated Plus Maze Test

Animals were placed in the plus maze with a central area (10 cm long^*^10 cm wide), two open arms (50 cm long^*^10 cm wide) and two closed arms (50 cm long^*^10 cm wide^*^40 cm high). This maze was 50 cm above the ground. Animals were free to explore the 4 arms for 5 min. All of the movements were recorded by the overhead camera. The travel time and number of entries into the open/closed arms were analyzed by ViewPoint Video Tracking Software (ViewPiont Behavior Technology, Lyon, France).

### Sucrose Preference Test

Animals were housed individually in cages, and provided with a bottle of water and a bottle of 1% sucrose solution. The position of the two bottles was switched to reduce the bias for place preference after 24 h. At the end of 48 h, bottles were removed and the liquid consumption was recorded. The percentage of sucrose intake was calculated as sucrose intake/total fluid intake ^*^ 100%.

### Forced Swimming Test

Animals were placed in a plexiglass cylinder (20 cm diameter × 60 cm height), filled with 30 cm of water maintained at ~25 ± 1°C and were forced to swim for 15 min to habituate. The next day, animals were placed in the water again for 5 min, and all of the movements were recorded by the overhead camera. The immobile time was assessed by ViewPoint Video Tracking Software (ViewPiont Behavior Technology, Lyon, France).

### Western Blot

After being deeply anesthetized by sevoflurane, animals were decapitated and hippocampus tissue was collected on ice. Protein lysates were prepared as previously described (29). Then, 50 μg protein was loaded and separated by a 15% SDS-PAGE gel, and then transferred to a PVDF membrane (Millipore, Billerica, MA, United States) at 200 mA for 2 h. After incubation in 1% gelatin solution for blocking for 1 h at room temperature, rabbit anti-BDNF (Santa Cruz Biotechnology Cat# sc-546, RRID:AB_630940), rabbit anti-p75 (Abcam Cat# ab8874, RRID:AB_306827), rabbit-anti-sortilin (Abcam Cat# ab16640, RRID:AB_2192606), mouse anti-MAP2 (Boster Cat# BM1243), rabbit anti-synaptophysin(SYP) (Proteintech Cat# 17785-1-AP) and mouse anti-GAPDH (CMCTAG Cat# AT0002) were applied to the membrane at 4°C overnight. Then the membrane was incubated with HRP-conjugated goat anti-rabbit IgG (Sigma-Aldrich Cat# A0545, RRID:AB_257896) or goat anti-mouse IgG (Sigma-Aldrich Cat# A9044, RRID:AB_258431) at room temperature for 1 h. The immunoreactivity of the proteins on the membrane was detected with an enhanced chemiluminescence kit (Millipore Cat# WBKL S00 50) and x-ray film (Carestream, United States). The band intensity was quantified using Image J software (NIH, Bethesda, MD, United States).

### Immunohistochemistry

After being deeply anesthetized by overdose chloral hydrate (400 mg/kg), animals were cardiac perfused with 100 ml normal saline and followed by 300 ml 4% ice-cold paraformaldehyde. The brain was harvested and post-fixed in 4% paraformaldehyde at 4°C overnight. Then the brain was dehydrated in 30% sucrose in phosphate buffer saline (PBS). The hippocampus region of the brain was sliced in a cryostat (CM1950, Leica Biosystems, Germany). Sections were washed in PBS and rinsed in 3% H_2_O_2_ for 30 min to remove endogenous peroxidase. Then, the sections were blocked by 5% BSA containing 0.01% Triton X-100 in PBS at room temperature for 2 h. Primary antibody anti-proBDNF monoclonal antibody which was generated by us and has been characterized previously was applied overnight at 4°C (29, 30). The sections were incubated in biotinylated goat anti-mouse immunoglobulin (Jackson ImmunoResearch Labs Cat# 111-065-003, RRID:AB_2337959) and followed by an ABC kit (Vector Laboratories Cat# PK-4000, RRID:AB_2336818). The glucose oxidase-DAB-nickel method was used for visualization (31). Finally, all of the sections were transferred onto gelatin-coated slides and dehydrated. The slides were cover-slipped with neutral balsam (ZSGB-BIO Cat# ZLI-9555) for visualization by optical microscopy (BX53, Olympus Corporation, Japan).

For confirmation of the Ab-proBDNF injection site, we performed the immunohistochemistry procedure mentioned above except for the application of the primary antibody.

### Golgi Staining

The staining procedure was carried out using an FD Rapid GolgiStainTM kit (FD Neuro-Technologies Cat# PK-401). In brief, animals were deeply anesthetized by sevoflurane and decapitated. The brain was collected and rinsed in Golgi–Cox solution, a mixture of solution A and solution B, and kept for at least 2 weeks in the dark. Then the tissue was kept in solution C for cryo-protection for 3 days. The brain was sliced into 150 μm sections by a cryostat (CM1950, Leica Biosystems, Germany). The hippocampus sections were transferred onto gelatin-coated slides. A mixture of solution D and solution E was used to visualize the neuronal architecture. After dehydration, the slides were cover-slipped with neutral balsam for visualization by optical microscopy (BX53, Olympus Corporation, Japan). Dendrite branches were traced by Image J software (NIH, Bethesda, MD, United States) with the NeuronJ plugin (32). Then dendritic length and spine density were calculated. Neuronal arborization was analyzed by counting the number of crossings by dendrites of concentric circles originating at the soma with increasing radii of 20 mm, using the sholl analysis plugin in ImageJ (33).

### Stereotaxic Surgery and Drug Infusion

The surgery was performed according to a standardized protocol (34, 35). After induction with 5% sevoflurane in the anesthesia chamber, rats were placed in a stereotaxic apparatus (68025, RWD Life Science, China) with continuous 2.5% sevoflurane inhalation. Two stainless steel guide cannulas were implanted bilaterally with the cannula tips 1.5 mm above the dentate gyrus (DG) area (AP-4.2 mm; ML ± 2.5 mm; DV-4.5 mm) (36). The guide cannula was secured with dental cement anchored to the skull. Stylets inserted into each cannula to maintain patency until the rats were subjected to delivery of drugs. The injection needle was inserted into the guide cannula, with its tip located 1.5 mm beyond the end of the guide cannula. Next, 1 μl monoclonal anti-proBDNF (29) antibody (1 μg/μl) was injected into the DG area in each hemisphere on day 22. The same volume of IgG (1 μg, 1 μg/μl) (CMCTAG Cat# AT1596) or normal saline was injected bilaterally into the same sites in the control group. The same dose of regents was administered repeatedly on day 28. From day 29 to 34, a series of behavioral tests was performed. Then rats were killed, and the brain was harvested for identification of the injection site or further analysis.

### Statistical Analysis

Data are expressed as mean ± SEM. Statistical analyses were performed using an unpaired two-tailed Student's *t*-test, one-way analysis of variance or two-way analysis of variance where appropriate. *p*-values were accepted as significantly different at *p* < 0.05. The statistical program used was GraphPad Prism 6.0 (San Diego, CA, United States).

## Results

### Different Behaviors in the Rats With Anxiety-Like Phenotype and Rats With Depression-Like Phenotype

**Figure 1:**
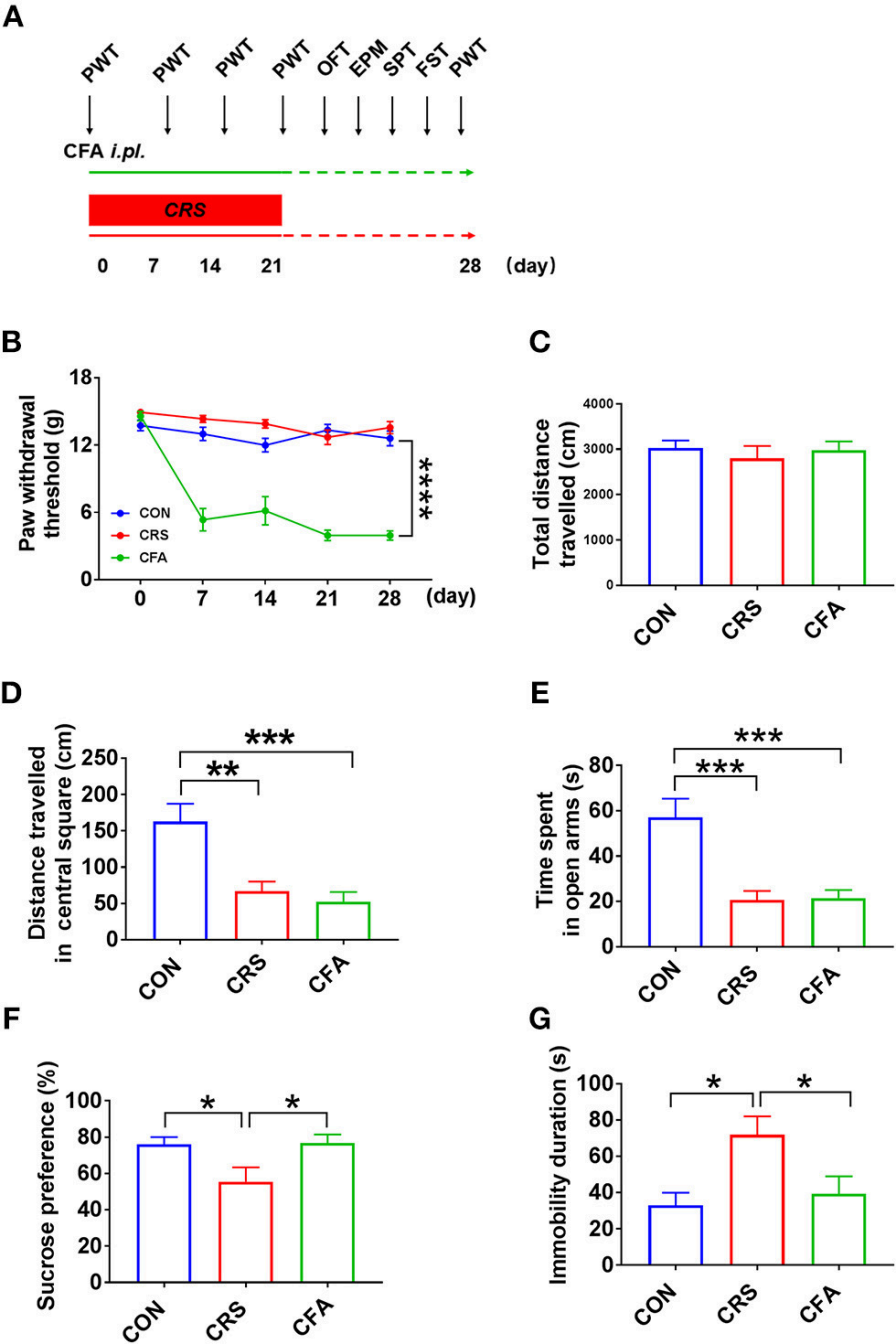
Behavior analyses of CFA-evoked anxiety-like and CRS-evoked depressive behaviors **(A)** Time schedule of behavioral tests of rats injected with CFA or subjected to CRS for 21 days. **(B)** A significant decrease in the PWT in CFA-injected rats at 7, 14, 21, and 28 days after injection. CRS did not affect PWT. A repeated-measured analysis of variance (ANOVA), ^****^p < 0.0001 vs. CON, *n* = 10 for each group; all graphs represent values in mean ± SEM. **(C–G)** CRS and CFA did not affect the total travel distance in the OFT **(C)**. Distance traveled in central square in OFT **(D)** and time spent in the open arms in the EPM test **(E)** were significantly decreased following CRS or CFA injection; meanwhile, CRS decreased sucrose intake **(F)** and prolonged immobility time in the FST **(G)**. CFA, complete Freund's adjuvant; CRS, chronic restraint stress; PWT, paw withdrawal threshold; OFT, open field test; EPM, elevated plus maze; SPT, sucrose preference test; FST, forced swim test. *N* = 9–10 in each group ^*^p < 0.05, ^**^p < 0.01, ^***^p < 0.001 vs. control group (one-way ANOVA). All of the data are presented as mean ± SEM.

Clinically, depression and anxiety result in different behaviors that can be substantiated via various behavioral tests: the self-rating anxiety scale, self-rating depression scale, or Hamilton depression scale tests. In rats, anxiety-like behavior can be induced by persistent inflammatory pain. It has been noticed that CFA intra-plantar injection rendered pain hypersensitivity as indicated by the decreased PWT (Figures 1A,B). Moreover, CRS-treated rats did not display mechanical hyperalgesia, whereas locomotor activity was not altered by CRS and CFA injection (Figure 1C). Both rats treated with CFA and those treated with CRS displayed decreased traveled distance in the central square in the OFT (Figure 1D) and reduced time spent in the open arms in the EPM (Figure 1E). In contrast, the SPT results showed that CFA-treated rats had comparable sucrose consumption with the controls whereas CRS-treated rats consumed less sucrose than both the controls and CFA-treated rats (Figure 1F). Similarly, in the FST, the immobility time of CRS-treated rats was longer than that of the controls and CFA-treated rats (Figure 1G). Thus, CRS-treated rats displayed anxiety- and depression-like behaviors, whereas CFA -treated rats only displayed anxiety-like behaviors.

### Different Granule Cell Morphology and Synaptic Integrity Changes Between Anxiety-Like and Depression-Like Behavior

**Figure 2:**
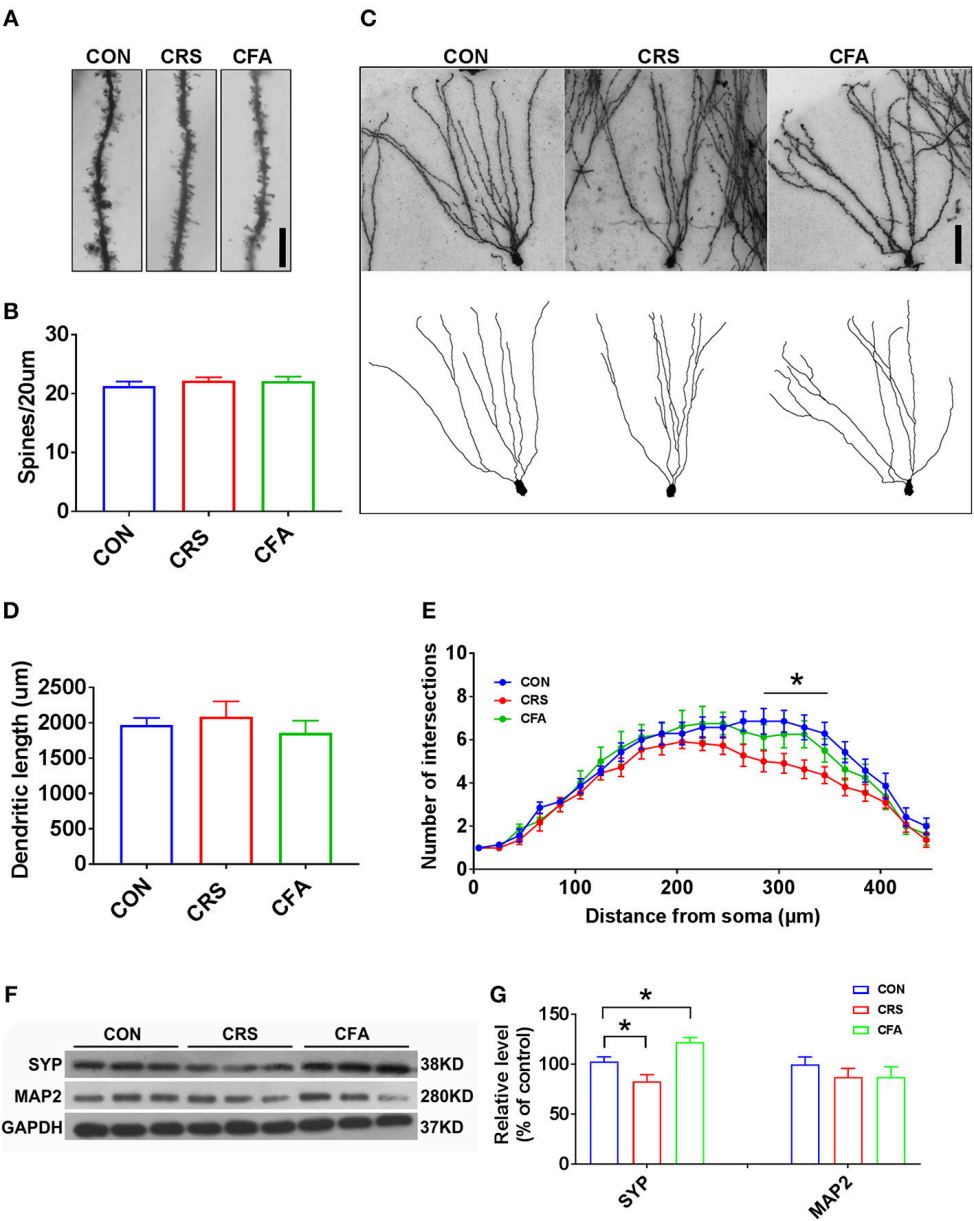
Granule cell morphology and synaptic integrity changes between anxiety-like and depression-like behaviors. **(A,B)** Representative Golgi-Cox staining images of dendritic spines and spine density in the hippocampal DG granule cells in CRS-treated rats, CFA-treated rats, and the controls. *n* = 10 for each group, bar = 10 μm. **(C)** Representative images of Golgi-Cox -stained granule cells in the DG area (top) and the reconstruction of its dendritic branches (bottom) from each group. Bar = 50 μm. **(D)** There were no significant effects of CRS and CFA injection on the dendritic length of the DG granule cells. **(E)** Sholl analysis of dendritic length in DG granule cells. CRS reduced dendrite intersection in the region 285–345 mm away from the soma compared with the control group. A repeated measures analysis of variance (ANOVA), ^*^p < 0.05 vs. CON, *n* = 10 for each group; all graphs represent mean ± SEM. **(F,G)** Expression of SYP and MAP2 in the hippocampus was evaluated by Western blotting. Semi-quantitative analyses of SYP and MAP2 expression were performed. Note that the expression of SYP was significantly reduced in CRS-treated rats but was increased in CFA -treated rats. DG: dentate gyrus; CFA: complete Freund's adjuvant; CRS, chronic restraint stress; SYP, synaptophysin; MAP2, microtubule-associated protein 2. ^*^p < 0.05, vs. control group (one-way ANOVA). All of the data are presented as mean ± SEM.

It has been reported that depression and anxiety involve synaptic changes (37–39). Therefore, we examined whether there were synaptic changes in these two animal models. Rats with depression-like phenotype and rats with anxiety-like phenotype had similar spine density and dendritic length of granule cells in the DG area compared with the controls (Figures 2A,B,D). However, sholl analysis showed a reduced complexity of granule neurons in rats with depression-like phenotype, and there was no difference in the dendritic arborization in rats with anxiety-like phenotype compared with the control group (Figures 2C,E). To assess the effect of CRS and CFA exposure on synaptic integrity, the expression levels of synaptophysin and microtubule-associated protein 2 (MAP2) in the hippocampus were measured. Synaptophysin was downregulated in the rats with depression-like phenotype but upregulated in the rats with anxiety-like phenotype. However, there was no significant change in MAP2 levels in the rats with anxiety-like phenotype and rats with depression-like phenotype as compared with the controls (Figures 2F,G). These results suggest that different morphologies and synaptic changes were responsible for depression and anxiety.

### Differential Expression of proBDNF and its Receptors in the Hippocampus in Rats With Anxiety-Like Phenotype and Rats With Depression-Like Phenotype

**Figure 3:**
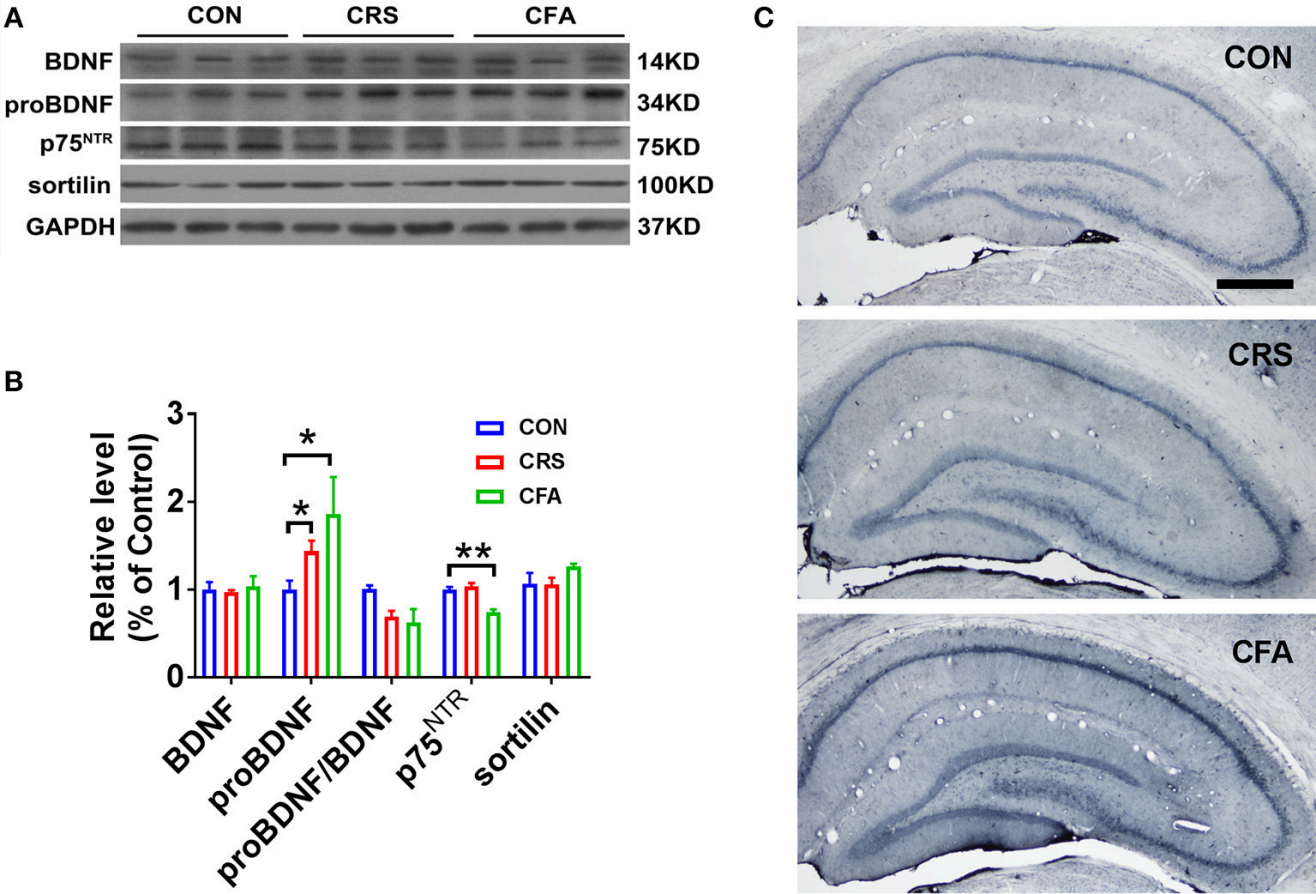
Upregulation of proBDNF and its signaling in rats with anxiety-like phenotype and rats with depression-like phenotype. **(A)** Representative Western blot, and **(B)** semi-quantitative analyses of mature BDNF, proBDNF, proBDNF/ BDNF, p75^NTR^, and sortilin in the hippocampus of rats with depression-like and anxiety-like phenotype. Note that the expression of proBDNF was increased significantly in the rats with depression-like phenotype and the rats with anxiety-like phenotype. **(C)** Immunohistochemistry of proBDNF in the hippocampus of rats with depression-like and anxiety-like behaviors. Note that intensive proBDNF was expressed in the hippocampus of the CRS rats and CFA rats. Bar = 100 μm. ^*^p < 0.05, ^**^p < 0.01, vs. control group (one-way ANOVA). All of the data are presented as mean ±SEM.

A recent study showed that chronic, unpredictable, mild stress upregulates proBDNF in the hippocampus of rats with depressive behavior (18). To further confirm the upregulation of proBDNF in depression and anxiety disorders, we examined the expression of proBDNF and mature BDNF in the hippocampus of these two rat models established in the present study. As shown in Figures 3A,B, proBDNF was found upregulated in the hippocampus. Interestingly, there was no significant change in BDNF levels in rats with depression-like phenotype or rats with anxiety-like phenotype as compared with the controls. These findings suggest that both depression and anxiety render the upregulation of hippocampal proBDNF. Surprisingly, the expression of hippocampal p75^NTR^ was not significantly changed in the rats with CRS-evoked depression-like behavior but was greatly down-regulated in the rats with CFA-evoked anxiety-like behavior. In addition, there was no significant difference in sortilin expression among the rats with anxiety-like phenotype, the rats with depression-like phenotype and the control group. Furthermore, proBDNF expression was intensely upregulated in the hippocampus of rats with depression-like phenotype and rats with anxiety-like phenotype (Figure 3C). These results suggest that depression and anxiety may have discrepant expression of proBDNF signaling.

### Intra-Hippocampal Injection of Ab-proBDNF Attenuated Anxiety-Like and Depressive Behaviors

**Figure 4:**
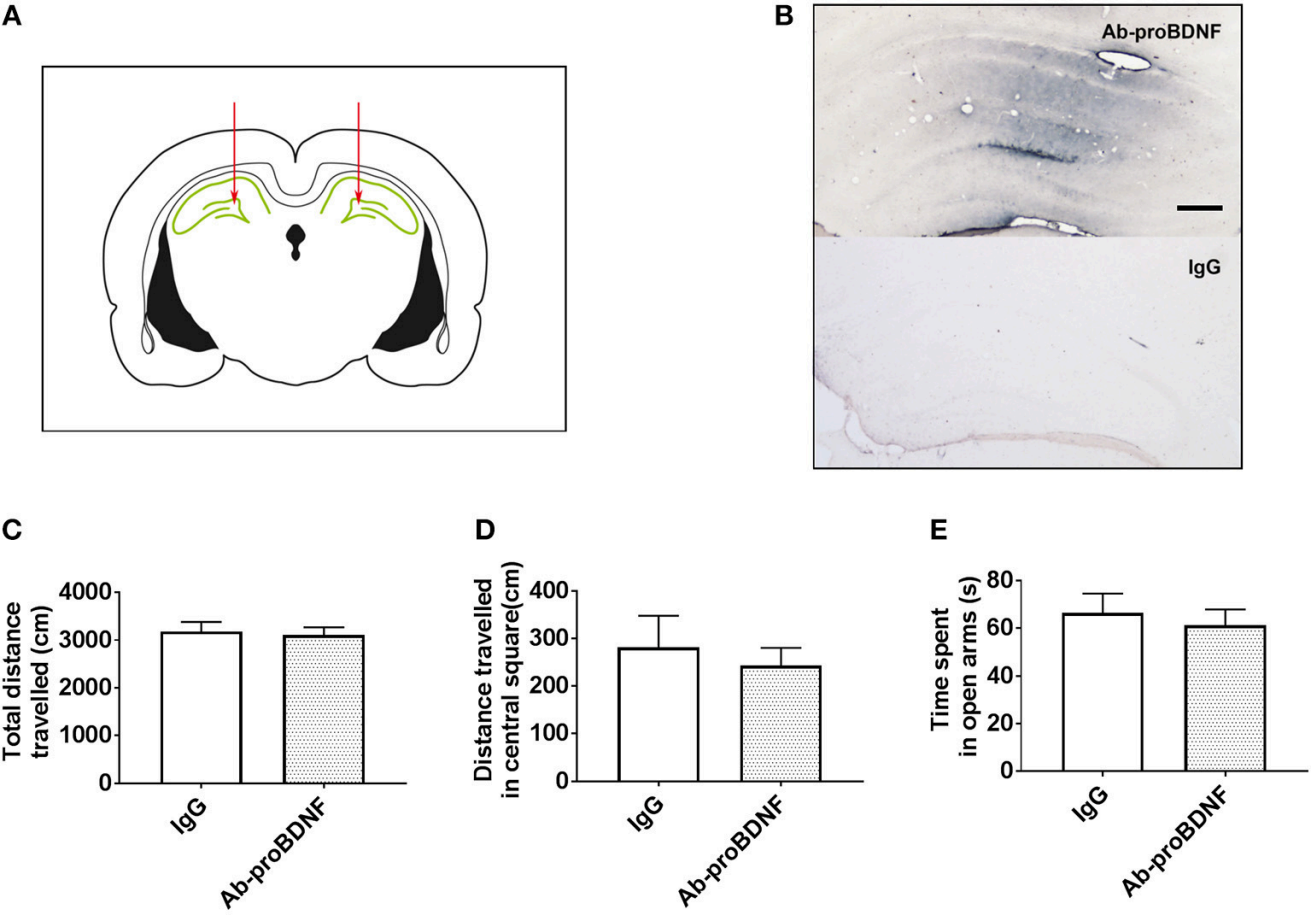
Hippocampal Ab-proBDNF injection and its effect on the anxiety-like behavior in normal rats. **(A)** Schematic representation of the antibody injection sites into the bilateral DG area through the cannula. **(B)** Representative immunohistochemistry images of Ab-proBDNF (top) or IgG (bottom) detection by the application of biotinylated anti-mouse IgG and the glucose oxidase-DAB-nickel visualization system 1 week after injection. Bar = 100 μm. Hippocampal Ab-proBDNF injection did not affect the total travel distance**(C)** or distance traveled **(D)** in the central square in the OFT, or the time spent **(E)** in the open arms in the EPM test. Ab-proBDNF: monoclonal anti-proBDNF antibody. *N* = 9–10 in each group. All data are presented as mean ± SEM.

The increased proBDNF in the hippocampus in the rats with anxiety-like phenotype and rats with depression-like phenotype may contribute to disease progress. In order to test this hypothesis, bilateral intra-hippocampal injection of monoclonal Ab-proBDNF (1 μg each side) was performed twice in the normal rats through the cannula. As shown in Figures 4A,B, intra-hippocampal injection of Ab-proBDNF was limited within the DG area, suggesting injection precision. Intra-hippocampal injection of Ab-proBDNF in the normal rats did not affect the total travel distance (Figure 4C) and the distance traveled in the central square in the OFT (Figure 4D). The EPM experiment also showed that injection of Ab-proBDNF into the hippocampus did not affect the time spent in the open arms (Figure 4E). These results suggest that intra-hippocampal injection of Ab-proBDNF did not change basal behavior.

**Figure 5:**
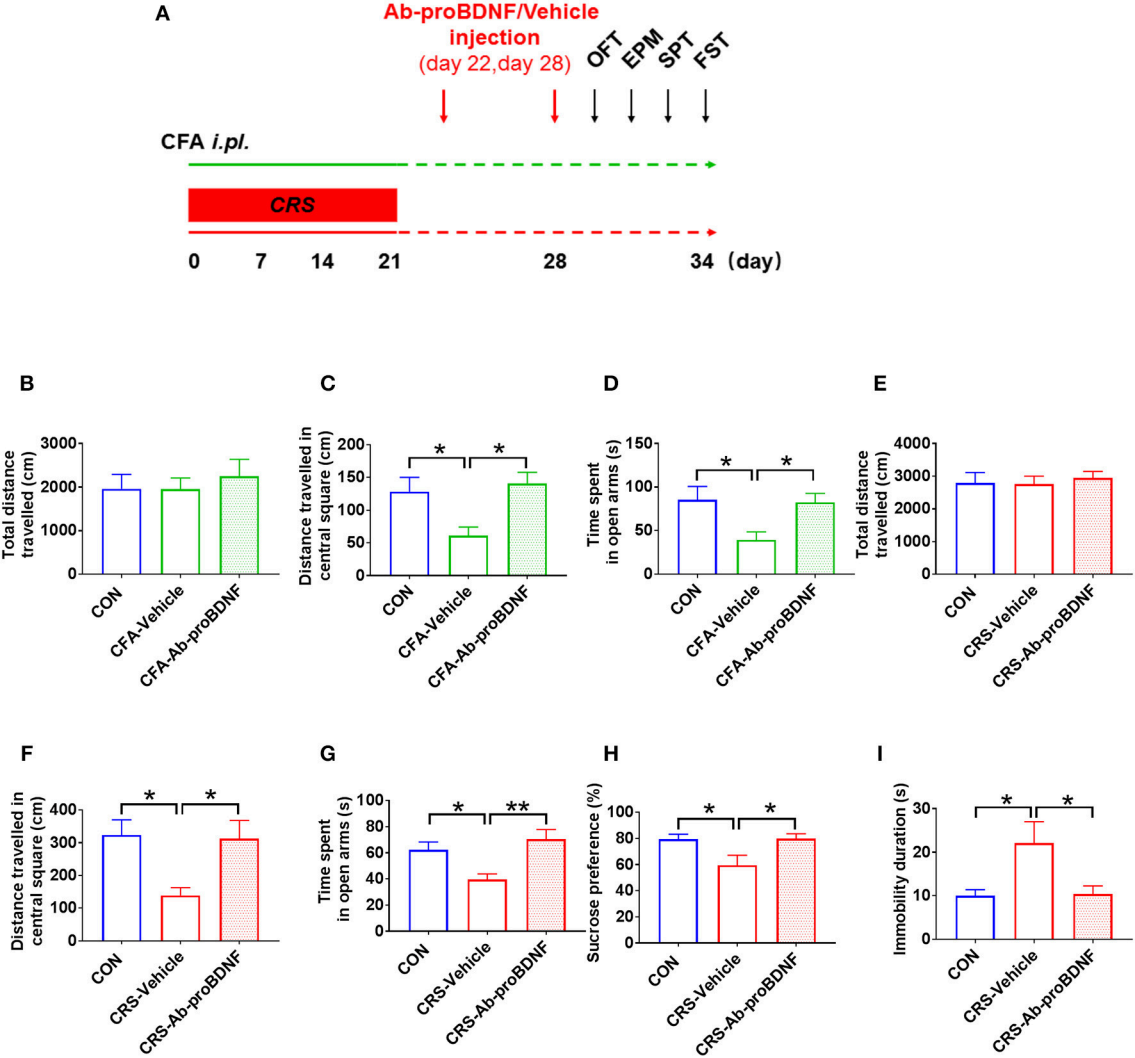
Hippocampal Ab-proBDNF injection ameliorated the anxiety-like and depressive behaviors. **(A)** Time schedule of behavioral tests and Ab-proBDNF injection in the CRS-evoked rats with depression-like phenotype and CFA-induced rats with anxiety-like phenotype. Hippocampal Ab-proBDNF injection did alter the total travel distance **(B,E)**, but reversed the decreased distance traveled in the central square in the OFT **(C,F)** and time spent in the open arms in the EPM test **(D,G)** in CFA-induced rats with anxiety-like phenotype and CRS-evoked rats with depression-like phenotype. Ab-proBDNF injection reversed the decreased sucrose intake in the SPT **(H)** and the increased immobility time in the FST **(I)** in CRS-evoked rats with depression-like phenotype. Ab-proBDNF: monoclonal anti-proBDNF antibody. *N* = 6–8 in each group. ^*^p < 0.05, ^**^p < 0.01, vs. control group (one-way ANOVA). All data are presented as mean ± SEM.

As shown in Figure 5A, bilateral intra-hippocampal injection of Ab-proBDNF (1 μg each side) was performed twice, on day 22 and 28, after CRS or CFA exposure. Intra-hippocampal injection of Ab-proBDNF reversed the reduction of distance traveled in the central area and the decreased time spent in the open arms in the rats with anxiety-like phenotype (Figures 5B–D). Similarly, as compared with the vehicle injection group, injection of Ab-proBDNF also inhibited the decreased distance traveled in the central square and the decreased time spent in the open arms in the rats with depression-like phenotype (Figures 5E–G). Moreover, neutralizing the endogenous proBDNF in the hippocampus by Ab-proBDNF injection greatly increased the sucrose consumption in the rats with depression-like phenotype compared with vehicle injection rats (Figure 5H). Finally, FST results showed that the immobility time was significantly lower in the Ab-proBDNF treatment group than in the vehicle treatment group (Figure 5I). Taken together, intra-hippocampal injection of Ab-proBDNF relieved the anxiety-like behavior and exerted an anti-depressive effect.

## Discussion

Anxiety disorders and depression are the two most prevalent mental disorders worldwide (40). In clinical practice, anxiety disorders and depression can overlap; many patients with depression having experienced anxiety disorders earlier in life. Moreover, around 50% of patients with depression are also diagnosed with an anxiety disorder (2). However, depression also displays different clinical manifestations than anxiety. For example, patients with depression move slowly with flattened or dulled reactions, whereas people with anxiety tend to be more keyed up. In addition, anxiety patients display fear about the future whereas depressed people are less likely to be fraught with worry about future events (41). All of these clinical manifestations suggest that these two distinct mental disorders are closely related, and may share some common mechanisms.

In the experimental rat models, both depressive and anxiety-like behaviors displayed the same behaviors in the OFT and EPM tests, which showed a decreased travel distance in the central square of the OFT and a decreased time spent in the open arms of the EPM test. However, depressive behavior can be distinguished from anxiety-like behavior through SPT and FST examinations in which rats with depression-like phenotype had less sucrose consumption and a longer immobility time (42). Like the anxiogenic effect of neuropathic pain (43, 44), CFA injection induced persistent inflammatory pain, which developed the anxiety-like behavior 3 weeks after CFA injection. It is consistent with the studies reported previously (27, 45). Notably, the chronic inflammatory pain may affect locomotor activity. However, the total traveled distance by rats with anxiety-like behavior in the OFT did not change, thus indicating that the reduction in traveled distance in the central square was not due to the effect of inflammatory pain on locomotor activity. Similarly to results found in previous studies (26, 46–48), rats with depression-like phenotype also exhibited lower sucrose consumption in the SPT and a longer immobility time in the FST. These findings further confirmed that there are some overlapping mechanisms between depression and anxiety in experimental animal models; these yet need to be unraveled.

Mental disorders are often accompanied with changes in dendritic arborization and spine density in the neurons of the hippocampus and prefrontal cortex; this is seen in both humans and rodents (49–51). In the present study, the dendritic complexity was significantly decreased in rats with depression-like phenotype, which is consistent with the results of previous studies (52–54). However, the dendritic length spine density was not changed in the rats with depression-like phenotype, and any difference in dendritic complexity, dendritic length and spine density was not found in the rats with anxiety-like phenotype. Moreover, in the rats with anxiety-like phenotype, synaptophysin, a marker for synaptic density found in the hippocampus, was increased, which is supported by previous reports (55, 56). In contrast, hippocampal SYP was decreased in the rats with depression-like phenotype. Whereas, there was no difference found in hippocampal MAP2 among all three groups. These findings suggest that the CRS results in hippocampal dendritic retraction and CFA slightly stimulates synaptogenesis in the hippocampus, providing the neurobiological substrates responsible for the different behaviors related to depression and anxiety.

The precursor of BDNF, proBDNF has been reported to regulate depressive behavior under chronic stress (18). proBDNF was upregulated in the hippocampus, neocortex and medial prefrontal cortex in rats with depression-like phenotype. In contrast, the expression of proBDNF was decreased in the nucleus accumbens in the rats with learned helplessness (19, 20), suggesting different expression patterns of proBDNF in different brain regions in the depression models. In the current study, the expression of proBDNF was also increased in the hippocampus of rats with depression-like phenotype. Furthermore, the expression of proBDNF signaling was also upregulated in rats with anxiety-like phenotype. Although intra-cerebroventricular injection of Ab-proBDNF could attenuate the depressive behavior (18), it is still unclear whether neutralization of the hippocampal proBDNF inhibits the anxiety-like and depressive behaviors.

In the present study, intra-DG injection of Ab-proBDNF in the control rats did not affect behavior as compared with the IgG control treatment, thus suggesting that the reduction of endogenous proBDNF in the hippocampus does not affect the behaviors under physiological conditions. This may be due to the low extracellular level of proBDNF, because only Ab-proBDNF can neutralize the extracellular proBDNF. In contrast to the lack of effect of Ab-proBDNF treatment on behaviors in control rats, Ab-proBDNF injection greatly inhibited the decreased sucrose consumption and increased immobility time in the FST. This indicates that hippocampal proBDNF contributes to the development of depression. Moreover, the intra-hippocampal Ab-proBDNF antibody also greatly protected against the anxiety-like behavior, as indicated by the EPM and OFT in both animal models. Collectively, these results indicate that hippocampal proBDNF is a common substrate that regulates depression and anxiety. Notably, in the present study, the injection site of Ab-proBDNF is mainly limited to the DG region. Previous studies have reported different alterations in the BDNF and proBDNF in CA1, CA3, and DG regions of the hippocampus within rodents with depression-like phenotype (20). Therefore, it would be greatly interesting to explore the role of proBDNF in CA1 and CA3 regions in depression and anxiety in a future study.

proBDNF exerts its biological effects through binding to its high affinity receptor p75^NTR^ and co-receptor sortilin. It has been reported that p75^NTR^ expression was increased in the rats depressed by unpredictable, chronic, mild stress (18). However, in the rats with CRS-evoked depression-like behavior, the expression of p75^NTR^ was not altered. This contrast may be due to the different paradigms used to induce depression. Nonetheless, the expression of p75^NTR^ was down-regulated in the rats with anxiety-like phenotype. This is consistent with the finding of a previous study, which showed that the deletion of p75^NTR^ resulted in anxiety-like behavior (57). In addition, there was no significant difference in sortilin expression in the rats with anxiety-like phenotype compared with the control rats. Recent studies showed that sortilin-knockout mice displayed anxiety-like behavior, suggesting the involvement of sortilin in anxiety (58). As there was no significant change in sortilin in the rats with anxiety-like phenotype in the present study, this indicates that sortilin may not contribute to the development of anxiety induced by chronic pain.

In conclusion, the present study showed that depression and anxiety have both distinct and overlapping behaviors, and morphological hippocampal changes in rat models. Furthermore, the increased hippocampal proBDNF played an important role in regulating both depression and anxiety. Inhibition of the increased proBDNF by antibodies might be a potential therapy to treat depression and anxiety.

## Author Contributions

FZ: conducted the study, contributed to data collection, data analysis, and manuscript preparation and revision. LeL and J-LW: contributed to the conduction of the study, data collection, and data analysis. Z-LH and SW: helped collect and analyze the data, and revise the manuscript. LiL and J-MX: helped design the study and analyze the data. X-FZ and C-QL: contributed to the experimental design and revision of the manuscript. R-PD and Z-YY: designed and interpreted the work, contributed to data collection, and data analysis, and assisted with manuscript drafting and revision.

### Conflict of Interest Statement

The authors declare that the research was conducted in the absence of any commercial or financial relationships that could be construed as a potential conflict of interest. The handling editor declared a shared affiliation, though no other collaboration, with several of the authors FZ, LeL, J-LW, Z-LH, LiL, SW, J-MX, C-QL, Z-YY, and R-PD, at the time of the review.
